# Editorial: Gut-lung interaction axis

**DOI:** 10.3389/fmicb.2023.1159629

**Published:** 2023-02-28

**Authors:** Jiang Pi, Guoliang Zhang, Gucheng Zeng

**Affiliations:** ^1^Guangdong Provincial Key Laboratory of Medical Molecular Diagnostics, The First Dongguan Affiliated Hospital, Guangdong Medical University, Dongguan, China; ^2^Institute for Hepatology, National Clinical Research Center for Infectious Disease, Shenzhen, China; ^3^Guangdong Key Laboratory for Emerging Infectious Diseases, Shenzhen Third People's Hospital, National Clinical Research Center for Tuberculosis, Southern University of Science and Technology, Shenzhen, China; ^4^Department of Microbiology, Zhongshan School of Medicine, Sun Yat-sen University, Guangzhou, China

**Keywords:** gut, lung, microorganisms, immunity, gut-lung interaction axis

Gastrointestinal diseases or disorders have been widely reported to accompany lung diseases. A wide range of microorganisms colonize in the epithelial tissue of the gastrointestinal tract and the lung compartment, and the mucous membranes of the intestine and respiratory tract provide a physiological and immune barrier against invading microorganisms. Recently, increasing studies have indicated that the gut microbe's unique composition, structure, and function and, consequently, their unique productions of metabolites and other components may effectively affect the occurrence and development of lung diseases. Thus, although the intestine and lung tissues are relatively far apart in their anatomic structure, they have similar cell origins and close physiological connections, which makes such extensive and intensive communications between the intestine and lung happen and sustain.

However, causal relationship between lung and intestinal disease are largely clinically and mechanistically uncharacterized, and, particularly, the exact microorganisms or microbial components that play a critical role in mediating health and diseases of intestine or lung through the gut-lung axis and their underlying mechanisms are still largely unclear. Therefore, identifying the exact microbial components and mechanisms that govern the gut-lung interactions is fundamentally important and may facilitate the development of new therapeutics against both lung and gastrointestinal diseases. Frontiers in Microbiology recently published a series of articles under the Research Topic “*Gut-lung interaction axis*.” This Research Topic contains four original articles exploring the potential linkages and underlying mechanisms for gut-lung interaction axis.

Xi et al. utilized fecal metagenome analysis to evaluate the association between gut microbiome signatures and disease progression in locally advanced non-small cell lung cancer (LA-NSCLC) patients treated with concurrent chemoradiotherapy (CCRT). They found that the baseline composition and functionality of gut microbiome might be associated with progression-free survival (PFS) rates in LA-NSCLC treated with CCRT, and, interestingly, the higher baseline microbiome diversity and the outcomes of CCRT might be modulated through bacterial metabolic pathways (Xi et al.). Moreover, their results indicated that the expression of antibiotic-resistance genes might play a role in disease progression, which therefore provide potential new information on the relationship between the use of antibiotics and therapeutic efficacy of CCRT in LA-NSCLC.

Xia et al. comparatively analyzed the lung microbiota and lung immune profiles in bronchoalveolar lavage fluid (BALF) derived from a total of 78 patients, including 21 patients with primary pulmonary tuberculosis (PTB), eight patients with newly diagnosed lung cancer (LC), and 49 patients with community-acquired pneumonia (CAP). They found increased bacterial α-diversity and richness in LC patients, and the CAP-associated pulmonary microbiota were significantly different between PTB and LC patients (Xia et al.). Additionally, BALF cytokine profiles were varied significantly and correlated with the key functional bacteria signatures in pulmonary microbiota of patients with PTB, LC, and CAP.

Zhu et al. explored the microbiome-driven pathogenesis mechanisms of pneumocystis pneumonia (PCP) in acquired immune deficiency syndrome (AIDS) patients. They found that human immunodeficiency virus (HIV) infection and PCP significantly altered the species compositions of both lung and intestinal microbiomes, and HIV infection significantly affected intestinal microbiome gene functions (Zhu et al.). They also found close correlations between different microorganisms and clinical indicators and their classification models that may have potentials to distinguish HIV+ from HIV– patients.

Mazzarelli et al. analyzed the association between gut microbiota and a combination of several clinical covariates to characterize the bacterial signatures associated with mild or severe symptoms during the SARS-CoV-2 infection. They found a significant greater proportion of Campylobacterota and Actinobacteriota at phylum level in SARS-CoV-2-infected patients affected who developed more severe diseases characterized by respiratory distress requiring invasive or non-invasive ventilation (Mazzarelli et al.). Their results showed that patients affected by SARS-CoV-2 with mild or severe symptoms displayed significantly different gut microbiota profiles, which can be exploited as potential prognostic biomarkers paving the new way to integrative therapeutic approaches.

In conclusion, these four articles introduced a series of interesting evidence advancing our understanding for the role of “*Gut-lung interaction axis*” in pathogenesis mechanisms of lung diseases and AIDS. However, more works are still needed to further explore the exact molecular events in “*Gut-lung interaction axis*” modulating the pathogenesis mechanisms of diseases, which may provide novel insights into diagnoses, prevention and treatments for pulmonary, intestinal, and potentially other systematic diseases.

## Author contributions

JP wrote the draft. GZh and GZe helped to revise the draft and were responsible for leading this work. All authors contributed to the article and approved the submitted version.

